# The Theory and Practice of Obstetrics

**Published:** 1889-04

**Authors:** 


**The Theory and Practice of Obstetrics, including Diseases of Pregnancy and Parturition, Obstetrical Operations, etc.** By P. CAZEAUX. Remodeled and re-arranged with additions and revisions by S. TARNIER. The eighth American edition. Edited and revised by ROBERT J. HESS, M. D., with an appendix by PAUL F. MUNDÉ, M. D., with chromo-lithographs. Lithographs and other full-page plates, and one hundred and seventy-five wood engravings. Philadelphia: P. Blakiston, Son & Co., 1002 Walnut street. 1889.

The present edition of Cazeaux and Tarnier's excellent work on Obstetrics is called the Student's Edition, for the reason that the publishers have issued the work in one volume, while the eighth edition, issued in 1886, was published in two volumes. The present edition is issued from the same plates, as its pages and the subject-matter are identical with the edition above alluded to.

It would be a superfluous task to review this work. It has been long ago included among the classes of obstetric science. It contains an inexhaustible fund of information, and the teacher, as well as student, will always refer to its pages in the search for facts and principles in studying this important department of medical science. The plan of the eighth edition has been greatly modified over that of previous editions. The subject is presented in seven principal parts, the first of these subdivisions being devoted to the Female Organs of Generation; the second, to Pregnancy; the third, to Labor; the fourth, to the Pathology of Pregnancy; the fifth, to Dystocia; the sixth, to Therapeutics; the seventh, to Obstetrical Operations. An appendix has been added to this edition from the pen of Dr. Paul F. Mundé, which includes the consideration of the (1) Hygiene of Pregnancy, Labor and the Puerperal State, (2) Posture in Obstetrics, (3) External Obstetric Manipulation, (4) Anesthetics, (5) Antisepsis in Obstetrics, (6) Puerperal Fever, (7) Puerperal Peritonitis and Cellulitis, (8) Lacerations of the Genital Organs, and their Influence on the Production of Subinvolution and Allied Pathological Conditions, (9) Primary Perineorrhophy, (10) The Diagnosis and Treatment of Extra-uterine Pregnancy, (11) Obstetric and Gynecic Jurisprudence.

A critical review of the work is unnecessary. The present edition is mainly fully up to the present state of obstetric science. When the present edition was issued in 1886, many subjects had not been as thoroughly investigated as at the present time. We are surprised that the publishers should not have noticed in this reprint the recent advances in ectopic pregnancy recently published by Lawson Tait in his admirable little work, which was reviewed in a late number of the JOURNAL; no other American writer is more familiar with these advances than the American editor. The same opinions on this subject are included in this reprint, as in the work as issued three years ago. A revolution in opinion and sentiment in this important and dangerous *mistake* in pregnancy, has taken place within the past three years. We are gratified to note the endorsement given to the value of antisepsis in midwifery, and the influence exerted by these measures in saving of material life. We think Dr. Mundé has demonstrated his ability in the selection of subjects for the appendix, and in handling the subjects so treated.

The work is a most valuable one. The library of the medical man is not complete without it, and we trust in the next edition the publisher will revise some portions of the work, bringing it in every part up to the present state of our knowledge, thus keeping it fully abreast with the advances made in the department of obstetrics.

